# Going Beyond Body Size in Fish Acoustic Communication

**DOI:** 10.1002/ece3.73547

**Published:** 2026-04-29

**Authors:** Lucia Yllan, Theresa Rueger

**Affiliations:** ^1^ School of Natural and Environmental Science Newcastle University Newcastle upon Tyne UK

**Keywords:** acoustic communication, anemonefish, bioacoustics, social rank, statistical analysis

## Abstract

In this response to Dr. Eric Parmentier's comments on our paper ‘“What Does the Anemonefish Say?”: Investigating *Amphiprion percula* 's Acoustic Behaviour’, we correct and clarify Dr. Parmentier's affirmations. Firstly, we address the statistical ‘problems’ by clarifying our statistical approach, highlighting the advantages of GLMMs in comparison with methods utilised by previous studies, such as correlation analysis. Secondly, we counter argument Dr. Parmentier's criticism of the inclusion of social rank as a variable alongside body size. Lastly, we highlight the need of an integrative framework and the use of new statistical approaches to deepen our understanding of fish acoustic communication.

## Abstract 2

This response addresses the methodological and conceptual concerns raised regarding our analysis of acoustic communication in anemonefish. We clarify our statistical framework, demonstrate that group structure and hierarchical relationships were appropriately accounted for, and explain why generalized linear mixed models provide a robust approach for evaluating acoustic variation. We further argue that incorporating social rank alongside body size is biologically necessary, highlighting the need for an integrative framework to understand acoustic signalling in hierarchical species.

## 1 Introduction

This letter responds to the comments made by Dr. Eric Parmentier (Parmentier 2026) regarding our study, ‘“What Does the Anemonefish Say?”: Investigating *Amphiprion percula* 's Acoustic Behaviour’, published in Ecology and Evolution (Yllan and Rueger 2025). While we appreciate the interest in our work, several interpretations presented in the commentary require clarification. This rebuttal addresses these points and provides supporting evidence for our original conclusions.

In this letter, we clarify key aspects of our analytical framework, demonstrate that group structure and hierarchical relationships were appropriately accounted for in our models, and explain why both body size and social rank were included as biologically meaningful predictors of acoustic variation.

Dr. Parmentier's comments fall into two main categories: statistical ‘problems’ and the inclusion of social rank and body size in the analysis. Thus, we will address his comments following this structure.

## 2 Statistical ‘Problems’

First, Dr. Parmentier argues that ‘Most importantly, the authors report no significant effect of body size on dominant or peak frequency after statistically removing the natural covariation between size and social rank. This approach effectively eliminates the biological signal expected in clownfish, whose hierarchies and acoustic scaling are tightly size‐dependent.’

This argument is misleading, since we tested the effect of body size alongside behaviour without removing the effect of social rank. Then, to further explore the effect of body size and social rank, we centred standard length which is a recommended method to account for collinearity and has been found to not alter statistical results (Dormann et al. 2013; Shieh 2011). Although it is true that we did not find a significant effect of body size on dominant or peak frequency when testing body size in the first part of our analysis, we did find effects of social rank and centred size in the frequencies in the second part of our analysis. If Dr. Parmentiers statements were true, we would expect the opposite results.

Second, Dr. Parmentier asserts that ‘[…] all individuals from all social groups were analysed together, without explicitly modelling the size structure or hierarchy specific to each group. In clownfish, rank categories do not guarantee size homogeneity (either within or across groups) and statistically centring body size further removes the natural covariance between rank and morphology.’

This statement is incorrect for several reasons. First, group structure was explicitly accounted for in our analyses by including group ID as a random effect in the models. Second, variation within ranks can be biologically meaningful: individuals may ascend in rank without immediate changes in body size, and rank can still be an important predictor of behavioural and social outcomes (see Rueger et al. 2022 for an experimental study showing behaviour changes immediately following rank ascension in *A. percula* ). Finally, centring body size does not remove the covariance between size and rank, it only changes the reference point to improve interpretability and reduce collinearity while the relationship itself remains intact.

Dr. Parmentier further claims that ‘In size‐based hierarchies such as those of anemonefish, submissive behaviours are almost exclusively performed by the smallest individuals in the group, whereas aggressive behaviours are typically produced by the largest individuals. As a result, each behavioural category is intrinsically associated with a distinct size class, which mechanically shifts the mean acoustic values attributed to each behaviour.’

This is also inaccurate. In our study, we explicitly tested interactions between size and behavioural category and included group identity as a random effect, ensuring that behavioural differences were not analysed independently of size. Therefore, our analyses do not introduce the biases suggested by Dr. Parmentier.

Regarding his recommendation that ‘it would be preferable to normalise acoustic parameters by body size. Without such correction, the observed variations are likely to reflect mainly the effect of body size rather than the behavioural context itself.’ We note that including body size as a predictor in a generalised linear mixed model (GLMM) effectively accounts for mechanical scaling without requiring additional normalisation. Hence, presenting the lack of normalisation as a methodological problem is misleading, and no evidence is provided that the observed effects are due solely to size differences rather than behavioural context.

The use of GLMMs in our study addresses statistical limitations present in previous studies, many of which relied on correlation analyses (Colleye and Parmentier 2012; Colleye et al. 2009). Correlation analyses are limited in that they only capture linear relationships, cannot account for confounding variables, cannot model causality, and cannot handle hierarchical or repeated‐measures data. By contrast, GLMMs allow us to model a response variable as a function of predictors while accounting for: non‐normal response distributions, random effects (such as repeated measures or nested data), multiple covariates simultaneously, non‐linear link functions, effect sizes and statistical significance of predictors. Notably, some previous studies (Colleye et al. 2011; Colleye and Parmentier 2012) pooled vocalisations per individual, removing intraindividual variation, which further highlights the advantages of our GLMM‐based approach in accurately capturing both size‐ and rank‐related variation in acoustic signals.

Another study employing mixed models similarly failed to detect a significant relationship between body size and acoustic frequency in anemonefish vocalisations (Hely 2022). Importantly, the study emphasised that analysing single pulses, rather than averaging values per individual, allowed intraindividual variation in acoustic frequency to be retained. The author compared both methods and argued that this variation, which is lost when means are calculated per individual as in earlier work (see Colleye and Parmentier 2012, and Colleye et al. 2011), reflects biologically meaningful complexity in vocal production. This methodological consideration aligns with our own analytical approach and further supports the inclusion of intraindividual variation when assessing relationships between body size and acoustic frequency.

Therefore, the discrepancies between our results and those of previous studies regarding the effect of body size on acoustic frequency can largely be attributed to methodological differences. Our use of generalised linear mixed models (GLMMs) allows us to explicitly model body size as a predictor while accounting for random effects, repeated measures, and hierarchical structure, without assuming linearity or pooling across individuals. Thus, our analytical methods provide a more rigorous and nuanced assessment of the factors influencing acoustic frequency, explaining why our results differ from previous studies.

## 3 Inclusion of Size and Rank in the Analysis

Dr. Parmentier comments that: ‘From a functional perspective, comparisons of acoustic features should therefore be based primarily on body size rather than rank, since only body size captures the mechanical constraints governing sound production.’

While we agree that body size imposes important mechanical constraints on sound production, we disagree that it should be the sole basis for comparisons. Focusing exclusively on morphology oversimplifies the complexity of acoustic signals and neglects the significant role of social rank within hierarchies. Social rank has been shown to be a strong predictor for certain factors such as behaviour in hierarchical systems (Yllan et al. 2024; Dijkstra et al. 2023; Maruska and Fernald 2010; Piefke et al. 2021). Changes in social rank trigger alterations in hormonal expression (Maruska and Fernald 2010; Oliveira 2009), which in turn can modulate auditory sensitivity and affect how acoustic signals are perceived and produced (Maruska et al. 2012). Furthermore, in cichlids, dominant males have been observed to produce specific calls to attract females, calls that are not produced by subordinate males, highlighting a socially mediated pattern of signal production that cannot be explained by body size alone (Maruska et al. 2012). Thus, social rank should be considered in tandem with body size to fully uncover the biological significance of these sounds.

In addition to this, we believe that Dr. Parmentiers statement contradicts his previous comment, where he acknowledges that social rank contributes meaningfully to the acoustic information in anemonefish vocalisations: ‘[…] removing the natural covariation between size and social rank. This approach effectively eliminates the biological signal expected in clownfish, whose hierarchies and acoustic scaling are tightly size‐dependent’.

Even the examples posed by Dr. Parmentier himself emphasise that individual fish discriminate not only by body size but also by social cues such as dominant status (Parmentier et al. 2009) and sex (Myrberg et al. 1986), potentially using acoustic signals to do so. These findings further reinforce our position that both social rank and body size must be taken into account to fully interpret the information encoded in acoustic signals.

## 4 Conclusion

In this response, we have clarified the analytical framework underpinning our study, demonstrated that group structure and hierarchical relationships were explicitly accounted for, and shown that the methodological concerns raised do not reflect shortcomings in our statistical approach. We have also outlined why the inclusion of social rank is not only justified but biologically necessary when investigating acoustic communication in hierarchical species.

It is important to emphasize that earlier studies were pioneering in establishing the link between morphology and acoustic communication in fishes, and they remain fundamental contributions to the field. However, as statistical methodology advances and our understanding of the complexity of fish social systems deepens, it is necessary to re‐evaluate analytical approaches accordingly. Taken together, these considerations highlight the need for an integrative framework in the study of acoustic communication.

## Author Contributions

**Lucia Yllan:** writing – original draft (lead). **Theresa Rueger:** writing – review and editing (lead).

## Conflicts of Interest

The authors declare no conflicts of interest.

## Data Availability

Mendeley Data at https://data.mendeley.com/datasets/7m9v6hxzrr/1, under the title. ‘ *Amphiprion percula* vocalizations data’ (Yllan et al. 2024). DOI: 10.17632/7m9v6hxzrr.1.
